# The complete mitochondrial genome of the hybrid *Furong-crucian* [*Cyprinus capio Furong* (♀) × *Carassius auratus red var* (♂)]

**DOI:** 10.1080/23802359.2016.1192511

**Published:** 2016-07-12

**Authors:** Li Zou, Jinlong Wang, Ming Zeng, Mingqiu Liu, Dongwu Wang, Yuanan Wu, Chuanwu Li, Guomin Jiang, Li Liu

**Affiliations:** a Department of Science, Fisheries Research Institute of Hunan Province, Changsha, Hunan, P.R. China;; b Collaborative Innovation Center for Efficient and Health Production of Fisheries in Hunan Province, Changde, Hunan, P.R. China

**Keywords:** *Furong-crucian*, Mitogenome

## Abstract

The complete mitochondrial genome of the hybrid of *Cyprinus capio Furong* (♀)* × Carassius auratus red var.* (♂) was characterized first in this study. The total length of the mitochondrial genome of *Furong-crucian* was identical to the female parent as 16,581 bp, and the overall base composition was 31.87% A, 24.81% T, 27.56% C and 15.76% G, with a slight A + T bias. It contained 13 protein-coding genes (PCGs), 22 transfer RNA genes, 2 ribosomal RNA genes and 2 main non-coding regions (the control region and the origin of the light-strand replication). This study discovered the 99.3% sequence identity between the hybrid and its female parent, which confirmed the maternal inheritance pattern followed by the mitochondrial genome of the hybrid. However, the sequence alignment of mitochondrial genomes between the hybrid and its female parent revealed a total of 109 variable sites in 18 genes or regions, especially 32 sense mutations in 9 PCGs. The complete mitochondrial genome sequence of this hybrid *Furong-crucian* may provide an important dataset for further study in mitochondrial inheritance mechanism.

In animal evolution, polyploidization and hybridization are a powerful approach of inducing genetic and epigenetic alterations, leading to increased diversity and speciation (Barton 2008; Guan et al. 2015). Distant hybridization makes it possible to transfer the genome of one species to another, which results in changes in phenotypes and genotypes of the progenies. A population of the hybrid of *Cyprinus capio Furong* (♀) × *Carassius auratus red var.* (♂) was obtained by artificial hybridization breeding and the obtained hybrid fish has many advantages including high rate of male, fast growth, high disease resistance and fleshy delicate. The hybrid *Furong-crucian* of *Cyprinus capio Furong* (♀) × *Carassius auratus red var.* (♂) is widely cultivated hybrid fish and very popular in many provinces in China. In this study, we obtained the hybrid *Furong-crucian* of *Cyprinus capio Furong* (♀) × *Carassius auratus red var.* (♂) complete mitochondrial genome. For a better understanding of the genetic status and the evolutionary study, we focused on the genetic information contained in the complete mitochondrial genomes of the fish.

The hybrid *Furong-crucian* was sampled randomly from Fisheries Research Institute of Hunan Province, Hunan province of China, and was deposited in the Museum of Fisheries Research Institute of Hunan Province (No. 219). We present the complete mitochondrial genome DNA sequence of the hybrid *Furong-crucian* by the PCR-based method for the first time. The experimental and data analysis methods followed the previous study (Chu et al. 2013; Liu et al. 2015). The mitochondrial genome has been deposited in the GenBank with accession number KU146531. The mitochondrial genome which was 16,581 bp in length included 13 protein-coding genes, 2 ribosomal RNA (rRNA) genes, 22 transfer RNA (tRNA) genes and one control region (D-loop), Except for ND6 gene and eight tRNA genes (tRNA-Gln, Ala, Asn, Cys, Tyr, Ser (UCN), Glu and Pro), all other genes were encoded on the H-strand. Adjacent genes overlap by a total of 23bp in seven different locations from 1 to 7bp, and have spacers of a total of 70bp in 13 different locations from 1 to 33bp. Except the gene COX1 used GTG as the initiation codon, all Protein-coding gene of the mitochondrial genome contains the strand start codon ATG. Seven genes end with the complete stop codon TAA or TA, while the COX2, ND2, ND3, ND4 and Cytb genes terminate with an incomplete stop codon T, which are often found within the mitochondrial genomes of teleost fishes, are completed via posttranscriptional polyadenylation (Chu et al. 2013; Zhang et al. 2014). The length of 22 tRNA genes ranged from 67bp to 76bp. All tRNA genes could be folded into a typical cloverleaf structure except for tRNA-Ser (AGY), which lost the dihydrouridine arm and formed a simple loop with 12 unpaired nucleotides. The gene arrangement and transcriptional direction were the similar to those of the typical teleosts mitogenomes (Chu et al. 2013). In the base composition of mitogenome, there was 31.87% A, 27.56% C, 15.76% G, 24.81% T, and a slight AT bias of 56.68% occured.

The sequence alignment of mitochondrial genomes between the hybrid and its female parent revealed a total of 109 variable sites in 18 genes or regions, especially, 32 sense mutations in nine PCGs (ND1, ND2, COX1, COX2, ATP6, COX3, ND4, ND5 and CYTB). These sense mutations may have some influences in the function of each control region. The phylogenetic analysis (Figure 1) showed that the studied hybrid *Furong-crucian* was relatively more close to *Cyprinus capio Furong*, which was its female parent. All the species from Cyprinus genus were gathered into the same branch (Tamura et al. 2011). This result was in agreement with the conventional taxonomic relationship of these species.

**Figure 1.**
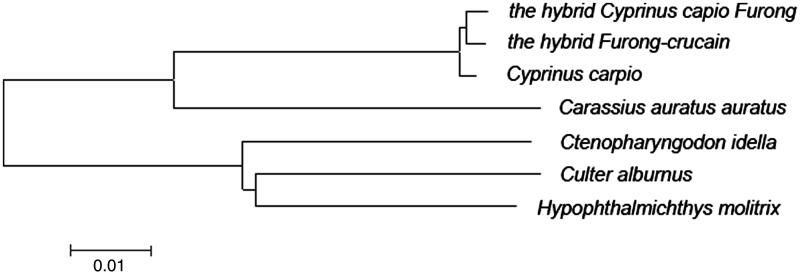
Phylogenetic relationship of The hybrid *Furong-crucian* complete mitochondrial genome DNA with other species. The phylogenetic tree was constructed by using the neighbor-joining method. The bootstrap confidence values shown at the nodes of the tree are based on 2000 bootstrap replications. Note: *the hybrid Furong-crucian*: KU146531; *the hybrid Cyprinus capio Furong*: KU146532; *Cyprinus carpio*: KU159761; *Carassius auratus auratus*: KU146528; *Ctenopharyngodon idella*: NC_010288; *Culter alburnus*: NC_013616; *Hypophthalmichthys molitrix*: NC_010156.
